# *Morpho* butterfly flashiness crucially depends on wing scale curvature

**DOI:** 10.1098/rsbl.2024.0358

**Published:** 2024-11-13

**Authors:** Juliana Sosa Espinosa, Doekele G. Stavenga, Casper J. van der Kooi, Marco A. Giraldo

**Affiliations:** ^1^ Groningen Institute for Evolutionary Life Sciences, University of Groningen, Groningen, The Netherlands; ^2^ Biophysics Group, Institute of Physics, University of Antioquia, Medellin, Colombia

**Keywords:** structural coloration, gloss, visual signalling, directionality, spectrophotometry

## Abstract

*Morpho* butterflies are widely known for their brilliant blue and flashy colours, which are produced by intricate wing scale structures. Not all species display a vibrant structural coloration; some are whitish or even brown. This suggests that there is considerable interspecific variation in wing scale anatomy, pigmentation and flashiness. As evidenced by numerous studies, the optical mechanism that creates the bright structural colours resides in the multilayered ridges of the wing scales, but the interspecific variation in flashiness has so far received little attention. Here, we investigate the wing components that influence the directional wing reflectivity. We therefore selected three species that greatly vary in colour and flashy appearance, *Morpho sulkowskyi*, *M. helenor* and *M. anaxibia*. Applying morphological analyses, (micro-)spectrophotometry and imaging scatterometry on wing pieces and individual wing scales, we demonstrate that wings with flat scales produce highly directional reflections, whereas wings stacked with curved scales scatter light into a wider angular space, resulting in a spatially more diffuse appearance. We thus find that the curvature of the wing scales crucially determines the directionality of *Morpho*’s visual display. We discuss how the visual ecology of *Morpho* butterflies and environmental conditions can drive the evolution of flashy visual displays.

## 1. Introduction

*Morpho* butterflies produce some of the most vibrant colours found in the animal realm. The genus *Morpho* comprises 30 species that vary in their wing coloration, sizes and shapes [1]. Some species showcase striking blue, iridescent colorations, whereas others are brownish or white [2–4]. The structural coloration resides in the tiny (approx. 100–200 μm) wing scales that consist of two chitin laminas, which are connected by trabeculae. The adwing (or lower) lamina of the scale that faces the wing substrate acts as an optical thin film reflector [5–7]. In addition, the abwing (or upper) lamina of the colourful scales of Morphos is an array of parallel ridges that consist of stacked lamellae, which together act as a multilayer reflector. In anatomical sections, the multilayer appears as a ‘Christmas-tree’ [5,6,8–10].

In addition to their overall blue coloration, the reflections of several species of *Morpho* are highly directional, which makes them appear flashy. As the multilayers are arranged in parallel ridges, they together act as a diffraction grating, so that the reflections of the wing scales are often directionally concentrated in a spatially narrow band [6,11–14]. Whereas many *Morpho* species exhibit a striking, flashy display, this is not shared by all species. The directionality of the visual appearance can dramatically vary, as some *Morpho* butterflies reflect light into a very wide angular space, thus creating a spatially almost uniform blue appearance. The morphological traits that determine the directionality of the butterflies’ visual display have so far only been studied in a few cases [15–17]. Here, we investigate how the shape of the scales can modulate the visual appearance of *Morpho* butterflies and their wings. Applying morphological analyses, (micro-)spectrophotometry and imaging scatterometry, we find that the curvature of the scales is a crucial factor that determines the spatial distribution of the reflections.

## 2. Methods

### (a) Specimens, photographs and microphotographs

For this study, we selected three *Morpho* species with prominent structural coloration, but with very different appearances: intact wings of male *M. sulkowskyi*, *M. helenor* and *M. anaxibia. M. sulkowskyi* and *M. anaxibia* were kindly provided by the Muséum National d'Histoire Naturelle, Paris, and *M. helenor* was purchased from commercial suppliers. To visualize the arrangement of the scales on the wing, we performed dark-field epi-illumination microscopy, using a Zeiss Universal Microscope (Zeiss, Oberkochen, Germany) with a Zeiss Epiplan objective (8×/0.2). To assess the shape of individual scales, we glued isolated scales to glass micropipettes with the scale’s longer axis parallel to that of the micropipette.

### (b) Spectrophotometry

Reflectance spectra of intact wings were recorded with an integrating sphere (Avasphere-50, Avantes, Apeldoorn, The Netherlands) using a Deuterium-Halogen lamp (AvaLight D(H)-S) and an AvaSpec-2048 spectrometer. A white reflectance standard (WS-2, Avantes) was used as a reference. Reflectance spectra of individual scales were measured with a microspectrophotometer, which consisted of an epi-illumination microscope equipped with an Olympus LUCPlanFL 20×/0.45 objective, coupled to the AvaSpec-2048 spectrometer through an optical fibre. The light source was a xenon arc lamp.

### (c) Imaging scatterometry

The hemispherical reflection pattern of wing pieces as well as of isolated wing scales was measured with an imaging scatterometer [14]. The wing pieces and individual scales, glued to a micropipette, were positioned at the first focal point of the ellipsoidal mirror of the scatterometer and illuminated with a narrow aperture, white-light beam. The diameter of the illuminated area was approximately 300 µm for wing pieces and approximately 13 µm for single scales. In the case of the isolated scales, five measurements, from the base to the tip of the scale, were recorded per sample. The spatial far-field reflection patterns were recorded with a FLIR BFLY-U3-23S6C-C camera (FLIR Integrated Imaging Solutions, Canada) and merged and processed using MATLAB R2021 and ImageJ.

## 3. Results

**Figure 1.**
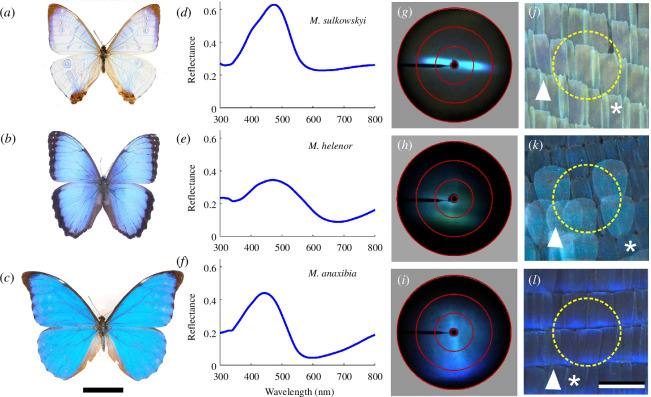
Three *Morpho* butterfly species that strongly differ in appearance and directionality of the visual display. (*a*, *d*, *g*, *j*) *M. sulkowskyi*. (*b*, *e*, *h*, *k*) *M. helenor*. (*c*, *f*, *i*, *l*) *M. anaxibia*. (*a–c*) Photographs; scale bar: 4 cm. (*d–f*) Reflectance spectra of intact wings measured with an integrating sphere. (*g–i*) Scatterograms of wing pieces; red circles indicate scattering angles of 5°, 30°, 60° and 90°. (*j–l*) Dark-field micrographs of intact wings with cover (arrowheads) and ground scales (asterisks); scale bar: 200  µm. Dashed yellow circles (diameter approx. 300 µm) indicate the illumination spot size applied in the scatterograms of panels (*g*–*i*).

The wings of male *M. sulkowskyi*, *M. helenor* and *M. anaxibia* show very different displays (figure 1*a–c*). *M. sulkowskyi* exhibits the strongest flashy appearance (figure 1*a*), the wings of *M. helenor* are mainly blue but have strongly contrasting black margins (figure 1*b*), whereas *M. anaxibia* displays a spatially more uniform blue coloration (figure 1*c*). Previous studies demonstrated that the blue coloration has a structural origin in the wing scales. Measurements of reflectance spectra of the wings with an integrating sphere show that the reflectance of *M. sulkowskyi* is higher than that of the other species and that it has an overall broadband background (figure 1*d–f*). This overall high reflectance is due to the lack of melanin pigmentation of the wing scales, which is considerable in *M. helenor* and *M. anaxibia* [6,18] and causes a strong modulation of the reflectance above 550 nm.

To further investigate the spatial distribution of the reflection, we performed imaging scatterometry on wing pieces to visualize the spatial spread of the wing reflections. We therefore illuminated a wing area, diameter approximately 300 µm, with a narrow aperture, white-light beam (figure 1*g–i*). The obtained scatterogram of a *M. sulkowskyi* wing shows a light distribution pattern that is restricted to a narrow spatial band (figure 1*g*). Quite different scatterograms are obtained of the wings of *M. helenor* and *M. anaxibia* (figure 1*h,i*). The latter two species exhibit spatially rather homogeneous reflection patterns, which suggests that the three species vary in their scale morphology.

**Figure 2.**
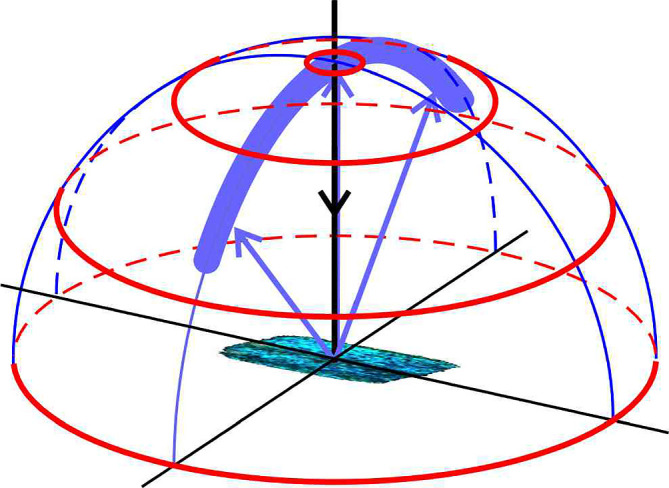
Diagram of light scattering by a *Morpho* wing scale. As the scale ridges are arranged parallel to the longitudinal axis of the scale, they together form a diffraction grating. The blue reflections of the multilayers of the ridges are restricted to a narrow spatial band, in a plane perpendicular to the axes of the ridges. The red circles, indicating scattering angles of 5°, 30°, 60° and 90°, correspond to the circles in the scatterograms of figures 1 and 3.

**Figure 3.**
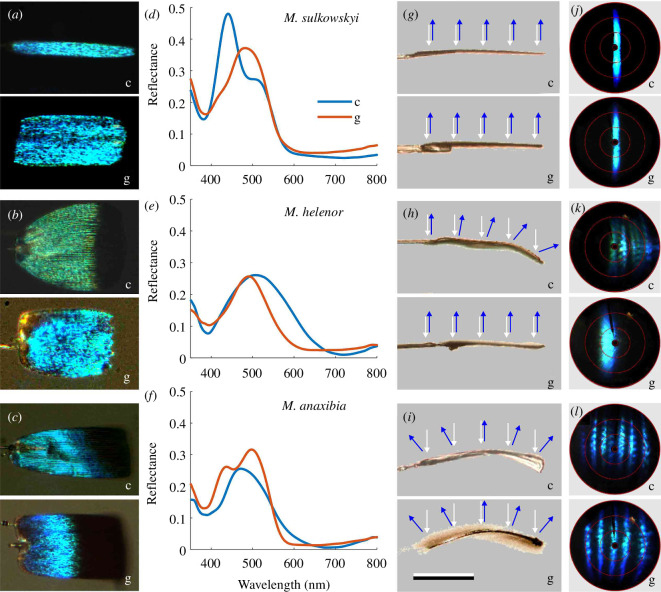
Reflection characteristics and shape of isolated scales. (*a–c*) Photographs of cover (c) and ground (g) scales of *M. sulkowskyi*, *M. helenor* and *M. anaxibia*. (*d–f*) Reflectance spectra of cover and ground scales. (*g–i*) Side views of the cover and ground scales; scale bar: 100 μm. Superimposed scatterograms of individual scales. The scales were illuminated at five locations (white arrows), and the reflected light (blue arrows) yielded scatterograms. (*j–l*) The joined scatterograms of the five cases obtained by photographing the individual far-field reflection patterns, which were subsequently superimposed.

Close-up micrographs of the wings indeed show rather species-specific scale lattices. Especially in the case of *M. anaxibia*, the localized reflections (blue edges) suggest strongly curved scales (figure 1*l*). Previous scatterometry on single *Morpho* scales showed that the multilayered ridges reflect light in a narrow spatial band [6,14] (see also the diagram in figure 2). In some species, the scales are wrinkled, which causes broadening of the spatial reflection bands, and also the reflections of the lower lamina’s thin film can be spatially displaced from the upper lamina’s multilayer reflections [6]. We therefore studied the reflection characteristics of both the cover and ground scales of the three chosen species (figure 3).

Wing scale curvature varies markedly among the three studied species and in one species (*M. helenor*) between scale types. A cover scale of *M. sulkowskyi* (figure 3*a*(c)) is much more slender than its ground scale (figure 3*a*(g)), but the reflectance spectra are restricted to the same blue wavelength range (figure 3*d*). Side views of both scale types reveal that they are nearly flat. Illuminating the scales very locally (spot diameter approx. 13 µm) at five different areas (figure 3*g*) yields virtually identical scatterograms. When the different scatterograms are superimposed they almost perfectly coincide, thus producing a very bright band (figure 3*j*). The cover scale of *M. helenor* is slightly broader than its ground scale (figure 3*b*(c)(g)) and the same holds for their reflectance spectra (figure 3*e*). The micrographs show that the cover scale is (half) curved, but the ground scale is flat (figure 3*h*). Accordingly, for the cover scale, the direction of the locally reflected light distinctly varies, as shown in the scatterograms when superimposed (figure 3*k*(c)). The superimposed scatterograms of the ground scales show a single, slightly broadened band (figure 3*k*(g)). Both the cover and ground scales of *M. anaxibia* are curved and have similar reflectance spectra (figure 3*f*). Local illuminations at various areas of the curved scales (figure 3*i*) yield displaced reflection bands in the scatterograms (figure 3*l*).

The scatterograms of single scales in figure 3 together explain the scatterograms in figure 1, obtained from the intact wings. In the case of *M. sulkowskyi*, the flat cover and ground scales are coplanar arranged, and the directional reflections of individual ground and cover scales (figure 3*j*) together create the narrow spatial band in the scatterogram of the wing pieces (figure 1*g*). On the intact wing of *M. helenor*, the cover scales reflect incident light in a broad spatial angle, but the ground scales reflect in a rather narrow band. On the wing of *M. anaxibia*, both cover and ground scales scatter light in a wide angular range, thus creating a diffuse reflection pattern (as documented in the scatterogram of figure 1*i*).

## 4. Discussion

The brilliant coloration of *Morpho* butterflies is commonly explained by the combination of the lower lamina that acts as a thin film reflector and the upper lamina’s stacked lamellae that act as a multilayer reflector [6,8,9,11]. The curvature is equally exerted on both the upper and lower laminae, as the joining trabeculae keep both scale layers attached one to another at about a constant distance. Hue and maximum reflectance may vary when comparing the coloration of different *Morpho* species, being the expected outcome of the variation in the multilayer arrangements, and thicknesses embedded in the nanostructures of the scales [19]. Our finding that the curvature of cover and ground wing scales determines the directionality of the display adds a hitherto largely neglected optical mechanism to the coloration toolkit of *Morpho* butterflies. Flat scales reflect light in a narrow angular space, creating a flashy appearance when the wing moves, whereas curved scales create a broad spatial reflection pattern. These findings dovetail with previous work on several species of Coliadinae [20]. The superposition of cover and ground scales, as well as the alignment of the scales in the wings, determines the signal directionality and the wing reflectance. An important additional observation is that the angular dependence of the display is mostly visible as a change in brightness (intensity) and less as a change in colour. This is because the underlying thin film and multilayer structures remain the same in curved and flat scales. In other words, under these conditions, the studied butterflies vary most in ‘flashiness’.

The visual appearance of a flashy butterfly is highly dynamic in time and space [21,22]. The wing beat frequency and the geometric organization of the signaller, receiver and light source are crucial factors of the butterfly’s visual appearance [23]. Flat wing scales further enhance the dynamicity of a fluttering butterfly’s appearance. In flight under the sun, a *M. sulkowskyi* butterfly will reflect brief and intense bursts of blue light. It is tempting to speculate that such bright, periodic flashes of light enhance long-distance visibility to potential partners, competitors and predators, analogous to how the flashing lights of a police cruiser attract the attention of humans [24]. Alternatively, a bright flash could saturate the receiver’s visual system, thereby limiting the observer’s ability to track the flashy object. Praying mantises appear to have more difficulty in tracking glossy than matte artificial prey items [25]. If butterflies suffer from a similar reduced ability to track a flashy object, this could explain why some species have evolved a more spatially homogeneous visual appearance. For example, the visual appearance of *M. anaxibia* will appear less variable than that of *M. sulkowskyi*, assuming a similar wing beat frequency. The perception of dynamic flash coloration probably depends on the speed of movement [26]. An alternative explanation for the observed differences in wing scale curvature is that they are a consequence of the illumination conditions. The illumination of a butterfly under a closed canopy is more spatially homogeneous (widefield) than under a clear sky. Highly directional visual effects will thus not come to fruition in closed canopy habitats, meaning that the visibility of a forest-dwelling individual may be higher when the visual appearance is spatially uniform. It is therefore likely that the perception of the observer and/or the habitat’s illumination conditions are drivers for the evolution of curved wing scales. Future research will reveal how the directionality of the visual display of *Morpho*’s and other animals with structure-based colours contributes to the visibility to partners, competitors and predators. Carefully designed behavioural experiments may be able to dissect the relative importance of changes in brightness, chromaticity, directionality and flicker frequency for this highly complex visual phenomenon.

## Data Availability

Spectrophotometry measurements are available as electronic supplementary material, which is available online [27].
